# PERSONAL DOSIMETRY USING MONTE-CARLO SIMULATIONS FOR OCCUPATIONAL DOSE MONITORING IN INTERVENTIONAL RADIOLOGY: THE RESULTS OF A PROOF OF CONCEPT IN A CLINICAL SETTING

**DOI:** 10.1093/rpd/ncab045

**Published:** 2021-04-06

**Authors:** A Almén, M Andersson, U O’Connor, M Abdelrahman, A Camp, V García, M A Duch, M Ginjaume, F Vanhavere

**Affiliations:** Medical Radiation Physics, Department of Translational Medicine, Lund University, Malmö, Sweden; Department of Radiation Protection, Swedish Radiation Safety Authority, Stockholm, Sweden; Medical Radiation Physics, Department of Translational Medicine, Lund University, Malmö, Sweden; Department of Medical Physics and Bioengineering, St James’s Hospital, Dublin, Ireland; The Belgian Nuclear Research Center, Boeretang, Belgium; Institut de Tècniques Energètiques, Universitat Politècnica de Catalunya (UPC), Barcelona, Spain; Institut de Tècniques Energètiques, Universitat Politècnica de Catalunya (UPC), Barcelona, Spain; Institut de Tècniques Energètiques, Universitat Politècnica de Catalunya (UPC), Barcelona, Spain; Institut de Tècniques Energètiques, Universitat Politècnica de Catalunya (UPC), Barcelona, Spain; The Belgian Nuclear Research Center, Boeretang, Belgium

## Abstract

Exposure levels to staff in interventional radiology (IR) may be significant and appropriate assessment of radiation doses is needed. Issues regarding measurements using physical dosemeters in the clinical environment still exist. The objective of this work was to explore the prerequisites for assessing staff radiation dose, based on simulations only.

Personal dose equivalent, *H*_p_(10), was assessed using simulations based on Monte Carlo methods. The position of the operator was defined using a 3D motion tracking system. X-ray system exposure parameters were extracted from the x-ray equipment. The methodology was investigated and the simulations compared to measurements during IR procedures.

The results indicate that the differences between simulated and measured staff radiation doses, in terms of the personal dose equivalent quantity *H*_p_(10), are in the order of 30–70 %. The results are promising but some issues remain to be solved, e.g. an automated tracking of movable parts such as the ceiling-mounted protection shield.

## INTRODUCTION

Occupational radiation protection in the medical sector continues to require attention, especially in interventional radiology (IR) where levels of exposure to the staff may be significant^(1–3)^. Therefore, individual assessment of radiation dose to staff is important when optimising radiation protection and certifying compliance with radiation dose limits. However, there are issues regarding individual radiation dose monitoring. Physical dosemeters exhibit significant uncertainties in the radiation energy and radiation dose rate ranges relevant in IR^(4)^. Other dosemeter properties, e.g. angular dependence, also further influence the uncertainties and can be a challenge for making accurate assessments of whole-body dose, equivalent dose to the skin and equivalent dose to the lens of the eye. Furthermore, due to the inhomogeneous radiation field, proper placement of the dosemeter on the body is crucial and several dosemeters may be required. These rather complicated procedures for wearing dosemeters are often not fully complied with by the staff. Additionally, optimisation of radiological protection requires radiation doses to be evaluated in detail after a limited number of procedures have been performed. The relatively low radiation doses from the procedures may challenge such measurement even more. In addition, it will be possible to estimate organ absorbed doses, e.g. equivalent dose to the lens of the eye using this computational method.

Simulations of staff radiation doses, without physical dosemeters, may be an alternative solution. To perform such simulations, data about the radiation source, the position of the c-arm, the use and position of radiation shielding and the position of the staff is required. The whole system including staff location and staff postures need to be modelled. In IR, the use of image acquisitions and fluoroscopy is extensive throughout a procedure and X-ray machine protocols and irradiation geometries vary significantly resulting in a complex radiation exposure situation. That is, the radiation exposure situation changes not only between different treatments but also during a treatment^(5)^. This requires a model of the irradiation situation that includes detailed information about the irradiation events coupled to the movement and body postures of the person of interest in a proper geometrical model.

In radiological protection, the Monte-Carlo technique simulating the radiation transport from a radiation source has been used widely for many years in various applications and numerous simulations in the field of IR have been performed, e.g.^(6–10)^. Phantoms, modelling humans, have been developed for use in various situations^(11)^. The least explored feature to date is the tracking of staff to determine position and postures^(12)^. Moreover, the complexity of the clinical exposure situation and its impact on prerequisites for the computational methods needs to be investigated.

This work aimed to explore occupational computational dosimetry in IR. More specifically to (i) explore the prerequisites for assessing effective dose to the operator during clinical IR procedures and (ii) to compare simulations and measurements during IR procedures using three Monte-Carlo codes for the simulations.

This work was part of the EC-funded project entitled ‘Personal Online Dosimetry Using Computational Methods” (PODIUM). The overall goal of the project was to develop a proof of concept of online personal dosimetry using calculations only. This was done by developing a method to be applied in the medical sector as well as industry. In this part of the project three particular Monte-Carlo codes were used to explore the possibilities of the proposed methodology. This paper covers the first investigations regarding IR whereby the novel indoor positioning system (IPS)^(12)^ was installed and tested in a clinic in an acute hospital in Sweden. The measurements were approved by the ethics committee (Number 2018/599).

## MATERIALS AND METHODS

The occupational dose, in this case personal dose equivalent, *H*_p_(10), to the physician performing the treatment—the operator—was assessed for one experimental set-up (Case A) and three clinical procedures (Case B-C-D). During the clinical procedures the dosemeters were worn over the lead apron, while *H*_p_(10) was assessed not including any lead apron into the simulations. A short description of the cases and required input data and simulations methods are provided below. The clinical cases are also described at the end of this section.

### Radiation source specification and geometry data available in the clinic required for the simulations

The exposure parameters for each irradiation event during a procedure were available in the DICOM standard radiation dose structure report (RDSR) derived from the workstation of the X-ray machine. The data include a timestamp for each irradiation event. The irradiation event could last over several 10th of seconds (image acquisitions) and several minutes (fluoroscopy). The tube voltage, filtration, angulation of the c-arm, beam size and output parameters, such as the air kerma area product, *P*_KA_, or the air kerma at the reference point, *K*_air_, were available as well as data about the size of the patient. The number of irradiation events per procedure may be extensive depending on the procedure type, in this work it varied from ~20 to 200 events. Irradiation events include fluoroscopy, where the operator typically stands near the patient during the event and does not move except for the arms and upper body. However, irradiation events also include image acquisitions where the operator may stand close to the patient, they may step outside the room or somewhere in between. The operator is also able to move away from the patient during the time it takes for one image acquisition.

### The simulation of personal dose equivalent

The simulations were performed using three Monte-Carlo photon radiation transport codes: MCGPU-IR^(13,14)^, PENELOPE/penEasyIR^(14,15)^ and MCNPx^(12,16)^. In order to accelerate the simulations, several variance reduction techniques and some geometry simplifications were implemented. PENELOPE/penEasyIR and MCNPx were run in a cluster of CPUs. PENELOPE/penEasyIR modelled the patient using a BOMAB-type phantom^(17)^ (BOttle Mannikin ABsorber) made of soft tissue. MCNPx modelled the patient using a BOMAB-type phantom for case B and for cases A and D—a prism was used in modelling the patient. In PENELOPE/penEasyIR and MCNPx simulations, the operator was not simulated and *H*_p_(10) was derived from the simulated photon fluence spectrum defined at a point of interest. In the case of MCGPU-IR, calculation time was reduced by using the computational power of commodity graphics processing unit (GPU). Both the patient and the operator were represented by anthropomorphic voxelised phantoms, they can be scaled differently (in three dimensions) so as to adapt their dimensions to the real values of both the patient and the worker. MCGPU-IR can provide the absorbed dose at a voxel level. The absorbed dose in specified organs in the patient and the operator, if the voxelised phantom is segmented, can be computed together with the effective dose. However, in this paper, since the other Monte- Carlo codes and the measurements only assess *H*_p_(10), the organ absorbed doses are not presented.

**Table 1:** **A summary of the exposure parameters and output in terms of *P***_**KA**_  **and *K***_**air**_  **for each case. The total treatment time, the use of the ceiling mounted shield and the used dosemeter are also presented**.

Case	A	B	C	D
Procedure	Experiment	Abdominal artery	Extremity artery	Renal artery
Irradiation event	1/1	115	19	186
No. of image acquisitions	—	17	7	10
Field size, cm^2^	835	457–1091	325–825	54–630
Tube voltage, kVp	79	70–86	66–82	68–85
Filtration, mm Cu	0.3	0.1 0.2, 0.3, 0.6, 0.9	0.3, 0.6, 0.9	0.2, 0.3, 0.6, 0.9
Angulation (+RAO/–LAO)	0/15	0–38	0	−35 to 26
*P*_ka_ Gycm^2^	4.1/4.2	163	0.06	10.5
*K*_air_ in reference point, mGy	17.6/18.0	679	0.3	200
Knife time, h:m	—	2:24	0:13	2:14
Ceiling shielding	No	Yes	No	Yes
Dosemeter	EPD, Mk2.3/TLD	Raysafe i2^a^	Raysafe i2	Mirion DMC 3000

^a^Measurements regarding one out of two operators.

The above-mentioned simplifications may affect the comparison with measurements and among MC codes. The features of the angiographic X-ray system and the exposure parameters were taken into consideration for the simulation of the primary beam. The output parameters, *P*_KA_ and *K*_air_, registered in the RDSR were used to normalise the simulated relative radiation doses to absolute radiation doses. The uncertainty of the output parameters was estimated by measurements using a calibrated semiconductor silicon detector (R100 Dose probe, Cobia, RTI, Mölndal, Sweden). The assessed differences were below 10%. Therefore an uncertainty of 10% (*k* = 2) was considered in the simulations.

### The determination of the position of the staff

In order to determine the position of the main operator, a 3D motion tracking system based on a depth camera (Microsoft Kinect v2) was used. The technical specifications of this camera have been explored^(18)^. The IPS used in this application has been described previously^(12)^.

The camera needs to have a proper view of the operator and the positioning is important and will vary from room to room. The final position chosen in this room was a position on the wall by the ceiling looking down at the table and operator. A process for the calibration was applied to derive a room coordinate system, which enables the operator position in relations to the radiation source to be assessed.

The timestamp of the irradiation event data as specified in the RDSR and the corresponding timestamp from the IPS were matched. The position of the operator is fixed at the beginning of each irradiation, assuming that the operator does not move during the whole irradiation event. However, in one case—case B described below—simulations taking into account the movement of the operator during an image acquisition lasting 7 s were performed. This type of adjustments of movement during an irradiation event was only performed once for this specific instance. The frequency of movement during a single irradiation event was not systematically investigated.

### A description of the experimental set-up and clinical cases

The investigation was performed at the vascular surgery clinic at the Skåne University hospital in a room equipped with a piece of modern angiographic equipment with a flat-panel detector (Siemens Artis Zee, Erlangen, Germany). The first case (A) involved a phantom in an experimental set-up and the other three cases (B-D) were clinical cases with personal dosemeters placed above the lead apron of the main operator. Exposure data and dosemeters used are summarised in Table 1, the cases are described below. The overall uncertainty in the assessment of *H*_p_(10) through measurements in a known X-ray beam was estimated to 20% (*k* = 2) for all dosemeters used.

**Figure 1:**
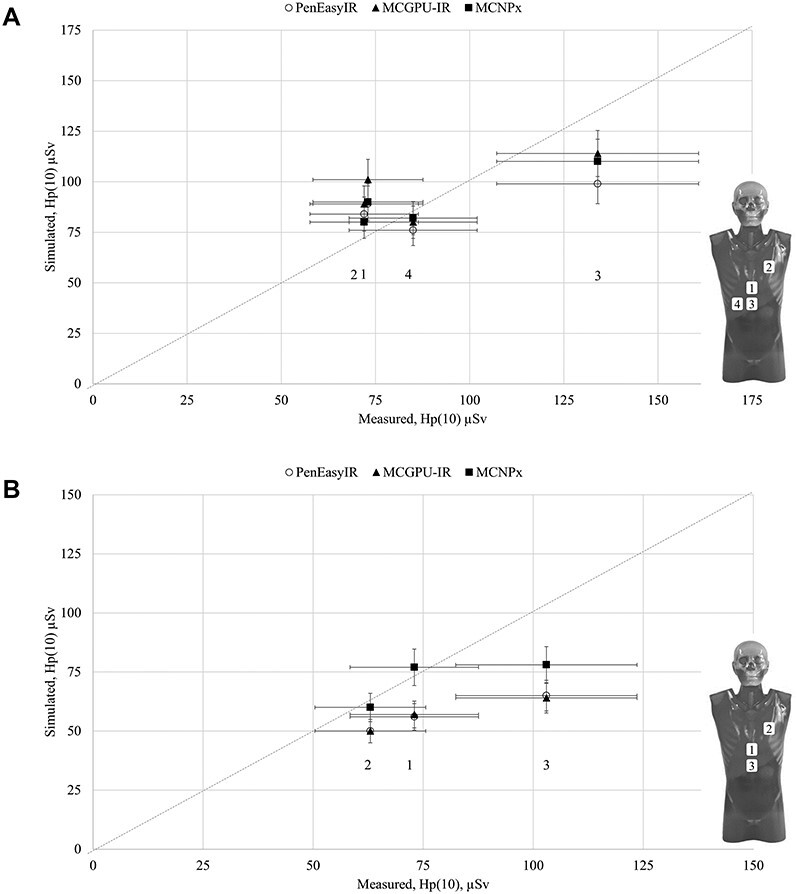
(a) Results from experimental set-up in case A (0°). Vertical error bars indicate the estimated uncertainty of 10% in simulations and horizontal bars indicate an estimated uncertainty of 20% in the measurements. The dashed line presents the theoretical line of identity between measurements and simulations. (b) Results from experimental set-up in case A (15°). Vertical error bars indicate the estimated uncertainty of 10% in simulations and horizontal bars indicate an estimated uncertainty of 20% in the measurements. The dashed line presents the theoretical line of identity between measurements and simulations.

In case A, a phantom modelling the patient (CIRS, Adult Male Phantom, Model No. 701, Norfolk, Virginia, USA) on the table and a phantom (Torso phantom CTU-41, Kyoto Kagaku, Japan) representing the operator was placed near the table and irradiated volume. No lead apron was placed on the phantom. Distances required in the simulations were measured manually. Measurements were performed for two different C-arm angles (0°, 15°), resulting in two separate measurements and simulations. The position of the dosemeters on the model operator is indicated in Figure 1a and b, respectively. The irradiation was performed using a fluoroscopy-only protocol (with no digital acquisition). The three Monte-Carlo codes were used in this case.

**Figure 2:**
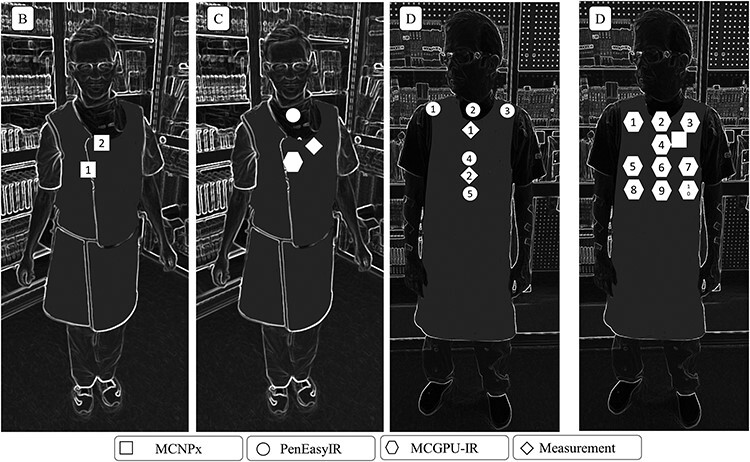
The position of the measurement and calculation in case B, C and D. In case B, the measurements are performed in the same position as the simulations.

Case B comprised a treatment of arteries in the abdomen. In this paper, one imaging acquisition (digital subtraction angiography) during which the operator moved away from the patient was examined and the possible consequences of the movement were investigated. The ceiling shielding was included in the simulations using an approximate position of the shield. The position of the dosemeters is presented in Figure 2 and the simulations using MCNPx were performed for these positions.

Case C comprised an investigation of a leg artery. No ceiling shielding was used during this treatment. This was a rather short investigation, including in total 19 irradiation events of which 7 resulted in a radiation dose to the dosemeter. The operator was positioned close to the patient for all irradiation events. Figure 2 indicates the placement of the dosemeter and points of calculation. The PENELOPE/penEasyIR and MCGPU-IR were used in this case.

In case D, a treatment of abdomen arteries was recorded including 186 irradiation events resulting in a relatively high radiation dose to the operator. The operator moved alongside the patient and left the room on some occasions. Ceiling shielding was used but simulations did not include ceiling shielding. The dosemeter positions are indicated in Figure 2 and simulations were performed using all three calculation methods. The data from these cases were compared to assess possible issues and differences.

## RESULTS

The assessed *H*_p_(10) for case A is presented in Figure 1a and b. The difference between the measured and simulated values varied between −45 and 45% and between simulations, the differences varied between –30 and 10%.

In case B, it could be seen from the positioning data that the operator moved during the image acquisition, and the distance for the final three frames was ~1–1.5 m from the patient, compared to their starting position which was much closer to the patient. The total radiation doses measured with the two dosemeters are included in Table 2, the average personal equivalent dose was 5 μSv. Simulated *H*_p_(10) using the operators starting position only, not taking the movement into account as done in the other cases, was 22 μSv. When the movement during the image acquisition as mentioned above was taken into account the simulated *H*_p_(10) was 7 μSv. This shows the importance of taking the movement into account.

**Table 2:** **Result of measurements and simulations for case B, C and D. *H***_**p**_ **(10) of the single points are presented and the simulated mean value with an uncertainty estimation of the points is presented in the right column**.

Case	Method	[#1, #2, #3….as in Figure 2] *H*_p_(10), μSv	Mean *H*_p_(10), μSv
B	Measured	[#1, #2] [4.2, 5.2]	5 ± 1
	MCNPx	[#1, #2] [20.3, 24.3]	22 ± 2
	MCNPx	[#1, #2] [6.2, 7.6]	7 ± 0.7
C	Measured	0.25	—
	PenEasyIR	0.31	—
	MCGPU-IR	0.19	—
D	Measured	[#1, #2] [7.5, 30]	18 ± 4
	MCNPx	35	35 ± 3
	PenEasyIR	[#1, #2, #3, #4, #5] [32, 47, 75, 50, 62]	53 ± 5
	MCGPU-IR	[#1, #2, #3, #4, #5, #6, #7, #8, #9, #10] [8.5, 8.5, 28.5, 18.5, 12.0, 20.8, 27.3, 17.3, 24.6, 31.2]	20 ± 2

In case C, the radiation dose to the operator is very low. The measured and simulated personal dose equivalents are presented in Table 2. The simulated values were 0.31 and 0.19 μSv. The personal dose equivalent measured was 0.25 μSv, outside the typical range. The percentage difference between the simulations is ~48%.

The result from case D is shown in Table 2, including values for each measurement and simulations point (Figure 2). The average values for the simulations were 53 (PENELOPE/penEasyIR) and 20 μSv (MCGPU-IR) to be compared with the value 35 μSv (MCNPx) and the average value for the measurement of 18 μSv. This shows the result of the inhomogeneous fields and illustrates the difficulties comparing simulations and measurements. The comparison between simulations and measurements are less relevant as the ceiling shielding used was not accounted for in the simulations.

## DISCUSSION

In this study, we have investigated the feasibility and challenges in calculating personal dose equivalent, *H*_p_(10), to the operator during IR for an experimental set-up and during clinical cases. *H*_p_(10) was assessed using three calculation tools and using personal dosemeters assessing the same quantity. The comparison indicates that differences between calculation methods are in the order of 10–100% and differences between simulations and measurements are in the order of the same magnitude. For comparison, the typical spread between active personal dosemeters (APD) is ~40–50%^(19)^. This study also shows the feasibility to use a phantom in the calculation. MCGPU-IR simulations are able to assess organ absorbed doses, and thus effective dose.

The phantoms used in this study were rigid. This study indicates the possible need to use flexible phantoms, mimicking the actual postures of the operator more in detail. This is important when assessing specific organ absorbed doses, e.g. absorbed dose to the skin or lens of the eye. These phantoms do exist, e.g.^(20)^ and the use together with a positioning system needs to be further explored. In addition, when comparing simulation results from the three Monte-Carlo codes, and also against measurements, it is worth mentioning that the used model of the patient can greatly affect the simulation results due to two different aspects; the complexity of the model (BOMAB or a voxelised phantom) and how it fits the actual dimensions of the patient.

One limitation in the simulations is that the shielding is not tracked and, thus, in most cases it is not included in the simulations. In one case in this study, the attenuation of a ceiling shield was added manually in the simulations. The information about the position and orientation of the shield in space needs to be an input variable in the simulations as this position may change. The X-ray tube could also be moved during the treatment, resulting in different isocenter positions. This movement has to be tracked as well which was a challenge for the current system and should be added to the model for the future.

This study showed that radiation field at the operator position is non-homogenous. This makes comparisons between measurement and simulations difficult and the radiation dose levels for some of the cases included in this study were also rather low, challenging the measurements. We also observed visually, that there is a possibility the operator may shield the dosemeter placed on their trunk with their arms during a treatment. More validations in both experimental set-ups and in the clinic are needed. However, the measurements are of importance for a proof-of-concept.

This study indicates that the proposed online dosimetry system can be a valuable tool for determining the radiation dose in IR and for optimising radiological protection. The system also has the potential to be used as a training and education tool. It is then desirable to show the link between protective measures and radiation dose levels to staff. This may be facilitated by also including video as a tool to make the measures visible.

## CONCLUSIONS

The obtained results indicate that the differences between simulated staff radiation doses, in terms of the personal dose equivalent quantity *H*_p_(10) are in the order of 30–70% with similar levels of differences between measurements and simulations. In the more complex investigated case including radiation shields, this figure increased to over 100%. The proof of concept PODIUM system was successfully installed in a busy interventional room and trialled during real clinical procedures. The three tested Monte-Carlo codes can be used in the proposed methodology and provide results with an acceptable speed and accuracy. The three MC codes have advantages and disadvantages. PENELOPE/penEasyIR and MCNPx are well established and validated codes, but at present they use very simple geometries to ensure a fast result. On the other hand, MCGPU-IR was developed in the framework of PODIUM and additional tests for its benchmarking are still needed. However, it provides absorbed doses to organs in simulation times that can be as short as 2 s (GPU use time) per simulated irradiation event. Finally, other issues such as software licence, cloud computing and interface to the end-user are important when choosing a code for an application suitable for a clinic. The results are promising but some issues remain to be solved, e.g. tracking of the movable shield during a procedure is needed.

The possibility to perform accurate Monte-Carlo simulated radiation dose estimations could be a valuable tool to overcome some of the issues that arise when using dosemeters. In addition, it would also provide the possibility to estimate organ absorbed doses, e.g. radiation dose to the lens of the eye or brain.
